# Anaesthesia for head and neck surgery: United Kingdom National Multidisciplinary Guidelines

**DOI:** 10.1017/S0022215116000384

**Published:** 2016-05

**Authors:** P Charters, I Ahmad, A Patel, S Russell

**Affiliations:** 1 Department of Anaesthesia, University Hospital Aintree, Liverpool, UK; 2 Department of Anaesthesia, Guy's and St Thomas’ NHS Foundation Trust, London, UK; 3 Department of Anaesthesia, Royal National Throat Nose & Ear Hospital, London, UK; 4 Department of Anaesthesia, Queen Elizabeth Hospital, Birmingham NHS Foundation Trust, Birmingham, UK

## Abstract

This is the official guideline endorsed by the specialty associations involved in the care of head and neck cancer patients in the UK. The anaesthetic considerations for head and neck cancer surgery are especially challenging given the high burden of concurrent comorbidity in this patient group and the need to share the airway with the surgical team. This paper provides recommendations on the anaesthetic considerations during surgery for head and neck cancer.

**Recommendations:**

• All theatre staff should participate in the World Health Organization checklist process. (R)

• Post-operative airway management should be guided by local protocols. (R)

• Patients admitted to post-operative care units with tracheal tubes in place should be monitored with continuous capnography. Removal for tracheal tubes is the responsibility of the anaesthetist. (R)

• Anaesthetists should formally hand over care to an appropriately trained practitioner in the post-operative or intensive care unit. (G)

• Intensive care unit staff looking after post-operative tracheostomies must be clear about which patients are not suitable for bag-mask ventilation and/or oral intubation in the event of emergencies. (R)

## Introduction

The anaesthetic and surgical team need to have a clear understanding about their respective roles in managing the ‘shared airway’. This will vary with the surgery and the anaesthetist's requirement to avoid airway compromise by way of gas exchange or soiling. A guaranteed airway from pre-operative ward care through to safe discharge must be considered as an essential duty of care for any institution undertaking surgery of this nature.

## Pre-operative assessment

Comorbidity and pre-operative assessment are considered elsewhere in the guidelines.^1^ Because of the ‘superficial’ nature of head and neck surgery, patients are less likely to be considered ‘unfit’ relative to those presenting for body cavity cancer surgery. One must be aware that this group of patients are prone to sepsis and multi-organ failure needing intensive care support. Such issues should be anticipated and discussed with the patient and relatives as part of the consent for surgery. Similarly, because many of the patients are elderly and with limited support at home, the implications of post-operative result and how the patient will be able to cope should be part of the decision to offer surgical treatment.

## General anaesthetic considerations

### World Health Organization (WHO) checklist

All theatre staff are recommended to participate in this initiative to ensure that teams work effectively and that the right patients get the right surgical procedure they have consented to. In addition, reference should be made to anticipated airway problems and ensuring the necessary equipment is available.^2^^,^^3^

### Monitoring requirements

The basic requirements for monitoring maintenance of anaesthesia and recovery are outlined in the Association of Anaesthetists of Great Britain and Ireland recommendations (4th edition, 2007) and advanced monitoring is usually only considered for long procedures or when excessive blood loss is a reasonable possibility.^4^

### Prophylaxis for thromboembolism is discussed elsewhere in these guidelines^1^

### Airway considerations

While patients presenting for head and neck surgery may have co-existent problems that could make airway management difficult (e.g. receding jaw, restricted neck movement, etc.), it is usually the size and site of tumour that causes concern. Any instrumentation needs to be judicious, including use of airway aids, in order that any problems with visualisation and/or airway soiling are not dramatically worsened. Patients with pharyngolaryngeal tumours frequently have residual food debris at laryngoscopy which may interfere with the view obtained especially for instruments with a limited field of vision. Contractures resulting from the previous treatment are common in patients with head and neck cancer. They may have obvious external deformities and restricted movements (e.g. limited neck extension). Rigidity and distortion of the oropharyngeal tissues can interfere with facemask ventilation and conventional laryngoscopy.

### Oxygenation

Maintenance of oxygenation is fundamental to airway management and techniques that extend the apnoeic window allow more controlled, less hurried and more careful, gentle instrumentation. This may reduce the deterioration in the airway following instrumentation and the subsequent difficulty in facemask ventilation which can lead to a ‘cannot intubate, cannot ventilate’ scenario.^5^ Traditional methods of increasing the apnoeic window involve spontaneous facemask ventilation with 100 per cent oxygen. Trans-nasal high-flow rapid insufflation ventilatory exchange or THRIVE delivered through a nasal high-flow oxygen delivery system has recently been shown to increase the apnoea time in head and neck patients including those with stridor to an average of 17 minutes. Trans-nasal high-flow rapid insufflation ventilatory exchange combines apnoeic oxygenation, continuous positive airway pressure and flow-dependent deadspace flushing and has the potential to change the nature of difficult intubations from a hurried stop–start process to a more controlled event, with an extended apnoeic window and reduced iatrogenic trauma.^6^

### Induction of anaesthesia

If a patient is already at risk of airway obstruction due to tumour bulk, then it is probable that they will be at greater risk following induction of anaesthesia, whether intravenous or inhalational. Even local anaesthesia is not without risk because severe airway obstruction precipitated by laryngospasm has occurred. In some institutions, ventilation is established prior to induction of general anaesthesia via temporary crico-thyroid or trans-tracheal access. (The latter is obviously preferable in patients with subglottic extension of a laryngeal tumour.) The use of muscle relaxant drugs to facilitate laryngoscopy in these cases is controversial because even if intubation conditions are improved this may be at the cost of greater risk of airway obstruction. Current practice has also been influenced by the introduction of many new intubation devices, very few of which have been reported in large series of head and neck cancer patients.

### Fluid management and blood loss

Many resections and free tissue transfers will not be associated with significant bleeding, though this is not necessarily true for tongue and mandibular resections where brisk bleeding may occur. Hypotensive conditions may minimise blood loss and haemodilution is practiced in some institutions with a view to improved blood flow in free flaps. Intra-operative haemoglobin and central venous pressure measurements help in monitoring the need for blood transfusion. Cardiac monitoring was used regularly in only 9 per cent of UK units in an audit in 2012.^7^

### Length of operative procedure

For lengthy operative procedures increased attention needs to be paid to the inevitable consequences of prolonged immobility, impaired homeostasis (associated with general anaesthesia) and the saturation of fatty tissue with anaesthetic agents. These equate to the need to protect from gravity-related pressure effects, thermal homeostasis, retention of urine and prolonged wake up time.

### Post-operative airway management

Currently there is widely diverse practice in terms of post-operative airway management of head and neck cancer patients. For example, at one end of the spectrum almost all free-flap reconstructions are managed with temporary tracheostomy whereas elsewhere, overnight ventilation followed by extubation the following morning is the expected norm. There are differences as to which patients warrant this level of airway protection and even as to suitability for delivery of such care by immediate return to the ward *vs* high dependency or intensive care. The need for advanced airway protection is to avoid airway obstruction due to haemorrhage or other surgical complication affecting the airway. Tracheostomy is an intervention with its own risks including inadvertent decannulation and is also associated with increased hospital stay. Overnight intubation may carry increased risk for patients with significant comorbidity. The relative decrease in senior and junior intensive care unit staff with no airway training may also condition local perceptions of relative risk.

**Recommendations**

• All theatre staff should participate in the WHO checklist process (R)
• Post-operative airway management should be guided by local protocols (R)

## Specific operative considerations

### The compromised airway

In the patient who presents with acute airway compromise the obvious option is to consider a tracheostomy under local anaesthesia. Even this may not be an easy option in the patient who is already desaturated, uncooperative and unable to lie flat. Because of the need to attend to the problem, there will be limited time for radiological imaging. Heliox mixtures may provide symptomatic relief, while further information is obtained, e.g. nasendoscopy to assess the airway objectively. Many of these cases will prove to have a laryngeal tumour, in which case surgeons generally prefer that tracheostomy is avoided. It may be possible to de-bulk the tumour once intubation is achieved, but experienced practitioners need to be involved if this is to be attempted.

### Tumour de-bulking to improve airway patency

Whether or not the patient presents as an emergency, there are two objectives. Firstly a biopsy will be taken for tissue diagnosis and secondly the tumour bulk will be reduced so as to minimise any likelihood of obstruction. Immediately after the procedure, the anaesthetist needs to confirm that the airway will be unobstructed (e.g. from a remaining tissue fragment acting as a ball-valve) and satisfactory from the point of view of bleeding.

### Formal tumour assessment for treatment planning (examination under anesthesia and biopsy)

This is the more usual situation where the risk of airway obstruction is considered less likely. The anaesthetist will usually have information about the lesion (e.g. photograph or clinical diagram) under consideration and ideally, shared visualisation of the lesion prior to induction.

### Tubeless anaesthesia

Ideally, any surgeon would wish to have an unrestricted view of the lesion to be operated on. In the case of laryngeal tumours, the most common compromise is to use a small diameter micro-laryngoscopy tube (6.0 mm ID or smaller). Other alternatives which allow a much less restricted field are: very narrow tubes used with gas exchanged by jet ventilation, a crico-thyroid airway (again usually with jet ventilation), *ad hoc* arrangements for repeated tube insertion and removal, and total intravenous anaesthesia with spontaneous respiration (usually also with local anaesthesia applied to the vocal cords). These alternatives tend to become more of a problem if the operative procedure is prolonged.

### Laser surgery

The risk of airway fires due to laser is low provided careful precautions including laser safe tubes are used. Post-operative haemorrhage and oedema risks mean that tracheostomy remains an important consideration in extensive resections.

### Free flaps

Attempts have been made to increase the success of free-flap anastomoses by medical means but there is no general consensus as to what if anything is efficacious. Doppler probes are available to monitor anastomotic vessel patency but are expensive and tend to be restricted in use to inaccessible sites, composite flaps (where skin colour may not reflect the deeper layer viability), continued arterial spasm risk and patients who have had previous radiation. Early return to theatre, however, in the event of compromise, may allow the flap to be salvaged if the blood flow can be restored.

### Management of surgical complications

Neck haematoma, flap failures, fistulas and airway management issues (e.g. re-establishment of a closed tracheostomy) are common reasons for a return to theatre. When patients are admitted to a post-anaesthesia care unit with tracheal tubes in place, continuous capnography monitoring is appropriate and their removal remains the anaesthetist's responsibility.^8^ It is important to be aware of the current state of the airway anatomy relative to the previous surgery and the time for healing. Severe bleeding is possible if major neck vessels are eroded. This sort of haemorrhage can arise suddenly and with little warning. Everyone involved needs to be acutely aware of what is needed by way of immediate measures (e.g. pressing on the neck in the event of a ‘carotid blowout’ or removing the skin clips in the event of a rapid expanding haematoma) *vs* the need to get to the theatre to attend to the problem directly. Proximity to the emergency theatres and kit available on the ward should be important considerations.

**Recommendation**

• Patients admitted to post-operative care units with tracheal tubes in place should be monitored with continuous capnography. Removal for tracheal tubes is the responsibility of the anaesthetist (R)

## Recovery from anaesthesia

### Emergence from anaesthesia phenomena

Commonly seen problems include transient hypertension, disorientation and/or agitation and shivering. Analgesic requirements tend to be less than for body cavity surgery, but this will not necessarily be the case in patients on moderate doses of opiates for pre-operative pain problems. Flap donor sites may have their own analgesic requirements.

### Immediate return to theatre from recovery

The most likely indications are bleeding and/or airway obstruction. The need for a covering tracheostomy may have been underestimated. Airway oedema can develop rapidly and is often precipitated by venous obstruction, posture change (e.g. allowing patients to lie down flat immediately prior to ward transfer) and Valsalva manoeuvres. Neck haematomas can be particularly deceptive because any associated airway oedema bears little resemblance to the apparent severity of neck swelling. If there is time it may be helpful to perform nasendoscopy prior to deciding how to anaesthetise for corrective surgical measures.

### High dependency and intensive care

Many head and neck surgery patients will be looked after in enhanced care by virtue of their comorbidity, the length of surgical procedure or the need to closely monitor the free flap. It is unusual for any patient to be ventilated post-operatively. Standardised handover forms are commonly used to summarise surgery and anaesthesia intra-operative events with a description of the resulting airway anatomical configuration and advisory options in the event of potential airway problems.

### Care of the tracheostomy

The Intensive Care Society has produced guidelines for the management of tracheostomy (and temporary tracheostomy in particular).^9^ Percutaneous and surgical tracheostomy is commonly used to help manage lower airway and aspiration problems in the general intensive care setting. Anticipated complications include bleeding, tube obstruction and accidental decannulation. Dealing with any of these issues commonly requires senior and experienced staff and they will frequently resort to conventional oral intubation to secure the airway prior to re-establishing the compromised tracheostomy, but oral intubation may not be feasible either because this is physically impossible (e.g. the post-laryngectomy patient) or because oral intubation would seriously jeopardise the surgical result (e.g. immediately after partial laryngectomy or major tongue resection). These situations can be very serious both because of the technical challenges posed and the limited time available for re-establishing the compromised airway. It is essential that anyone dealing with these situations must know what surgery has been performed and whether oral intubation is a feasible alternative.

### Enhanced recovery programmes (ERP) for head and neck cancer patients

An ERP can be formulated around the head and neck cancer patient's overall journey.^10^^,^^11^ Stratified introduction of interventions with simple early objectives may yield a positive impact on outcomes. These programmes have been shown to improve outcomes in patients undergoing major colorectal and gynaecological procedures, by reducing length of stay and 30-day morbidity. Extrapolation of these concepts to patients with head and neck cancer undergoing major resections and free-flap surgery may help in improving outcomes. Relevant pre-operative measures might include carbohydrate loading with carbohydrate drinks 1–2 days before surgery. Intra-operative goals include: directed fluid therapy using cardiac output monitoring to optimise fluid management; maintenance of normothermia and tight glycaemic control. In the post-operative phase, early enteral feeding is advocated.

**Recommendations**

• Anaesthetists should formally hand over care to an appropriately trained practitioner in the post-operative or intensive care unit (G)
• Intensive care unit staff looking after post-operative tracheostomies must be clear about which patients are not suitable for bag-mask ventilation and/or oral intubation in the event of emergencies (R)

### Key points

• The main difference between anaesthesia for major head and neck surgery and that for body cavity cancer is that because it is relatively superficial patients with greater comorbidity can be treated
• Overall care of the airway for these patients should be seen as an institutional responsibility where all the weakest points in care delivery are addressed
• Perioperative assessment should be comprehensive enough to make all airway issues predictable and suitably planned for
• Pre-oxygenation by trans-nasal high-flow rapid insufflation ventilator exchange (“Thrive”) significantly extends the window for tracheal intubation, making it less stressful and less traumatic
• Operatively “shared airway” working (between the surgeon and anaesthetist) should be seamless with anticipation of one another's requirements
• Post-operative airway issues can occur even with minor surgical procedures, again these should be anticipated and planned for
• Significant diversity exists in the expected post-operative care for major cases (mainly tracheostomy versus overnight intubation and extubation the following day)
• Staff caring for patients with tracheostomy and serious airway pathology must be aware of the special risks this implies, suitably trained and aware of the relevant guidelines
• Urgent airway issues need a planned response that takes into account local resource allocation and proximity between wards, theatre and HDU/ICU
• When urgent airway issues arise the institution must be able to match the response to the problem with appropriate seniority of expertise.
